# miR-520a-5p regulates Frizzled 9 expression and mediates effects of cigarette smoke and iloprost chemoprevention

**DOI:** 10.1038/s41598-022-06292-7

**Published:** 2022-02-11

**Authors:** A. J. Smith, P. Do, K. Sompel, A. Elango, M. A. Tennis

**Affiliations:** 1 Division of Pulmonary Sciences and Critical Care Medicine, School of Medicine, University of Colorado Anschutz Medical Campus, Aurora, CO USA; 2 Skaggs School of Pharmacy, University of Colorado Anschutz Medical Campus, Aurora, CO USA; 3 University of Colorado Denver AMC, 12700 E 19th Ave, RC2 Box C272, Aurora, CO 80045 USA

**Keywords:** Cancer prevention, Lung cancer, Non-coding RNAs, Mechanisms of disease

## Abstract

Expression of Frizzled 9 (FZD9) is critical to the activity of the lung cancer chemoprevention agent and prostacyclin analogue, iloprost. FZD9 is required in lung epithelial cells for iloprost to activate peroxisome proliferator activated receptor gamma (PPARG) and related anti-tumor signaling. We aimed to investigate which miRNA regulate FZD9 in the context of cigarette smoke exposure and iloprost treatment. We found that miR-520a-5p binds the FZD9 3’UTR in lung cell lines and alters activity and expression of FZD9 downstream targets. Cigarette smoke condensate (CSC) increases expression of miR-520a-5p, while iloprost decreases expression. Cancer promoting effects of a miR-520a-5p mimic were rescued with iloprost treatment, and effects of cigarette smoke were partially rescued with a miR-520a-5p inhibitor. Here we confirm miR-520a-5p as a regulator of FZD9 activity and a mediator of CSC and iloprost effects in the lung. Targeting miR-520a-5p could be an approach to restoring FZD9 expression and improving response to iloprost lung cancer chemoprevention.

## Introduction

Understanding the interaction of the lung epithelium with carcinogens and chemoprevention agents is critical to advancing interventions aimed at halting progression of premalignant lung lesions. We have identified the transmembrane receptor Frizzled 9 (FZD9) as a mediator of the effects of lung cancer chemoprevention with prostacyclin. FZD9 is a non-canonical WNT receptor in the lung that acts as a tumor suppressor rather than an oncogene^1^. When activated by the endogenous ligand WNT7A, FZD9 maintains a normal lung epithelium by signaling to the peroxisome proliferator activated receptor gamma (PPARG), an inhibitor of epithelial to mesenchymal transition^2^. While MEK5 and ERK5 have been identified as signal transducers in the FZD9/WNT7A pathway, most components of the pathway important for FZD9 activation and signaling in the lung are unknown^2^. Similar to activation by FZD9, PPARG is also activated by prostacyclin in lung epithelial cells, leading to anti-tumor signaling^3,4^. NSCLC cell lines have very low levels of prostacyclin, along with low expression of PGIS and the expected prostacyclin receptor, prostaglandin I2 (IP), and human NSCLC protein samples have reduced or absent PGIS expression^5,6^. Increasing prostacyclin by genetically overexpressing prostacyclin synthase (PGIS) or treating with the prostacyclin analogue iloprost decreases the development of lung tumors in urethane or cigarette smoke exposed mice^3,7–9^. The development of lung cancer is still inhibited in PGIS transgenic/IP knockout mice, suggesting that prostacyclin is acting independent of the IP receptor^7^. In vitro and in *vivo* modeling of prostacyclin in the lung epithelium have suggested FZD9 is part of the preventive signaling pathway^4,10^.

The effectiveness of lung cancer chemoprevention with iloprost in humans was demonstrated in a clinical trial in high-risk patients. In this phase II trial, current and former smokers were treated with six months of oral iloprost or placebo; former smokers treated with iloprost had reduced endobronchial dysplasia compared to placebo treated former smokers^11^. Current smokers had no benefit. FZD9 is required for iloprost activation of PPARG and transformed growth inhibition in vitro, implicating FZD9 as a potential membrane receptor for iloprost in the lung epithelium^4^. Iloprost and cigarette smoke condensate (CSC) have inverse effects on FZD9, where iloprost exposure increases and CSC decreases expression and downstream activity^10^. FZD9 expression could be both a marker of risk and an iloprost response indicator. Among former smokers in the chemoprevention trial who received iloprost, about half had improved histology, which could be due to a spectrum of response based on variable FZD9 expression^11^. This variability may help further characterize candidates for iloprost chemoprevention among high-risk patients. FZD9 is rarely mutated in lung cancer, suggesting that dysregulation of FZD9 in the lung epithelium occurs at the transcriptional or translational level. In this study, we identified miR520a-5p as binding to the FZD9 3’UTR and inhibiting FZD9 downstream activity and investigated its role in mediating the effects of iloprost and CSC on lung epithelial cells.

## Results

### miR-520a-5p inhibits activity of the FZD9 3’UTR

**Figure 1:**
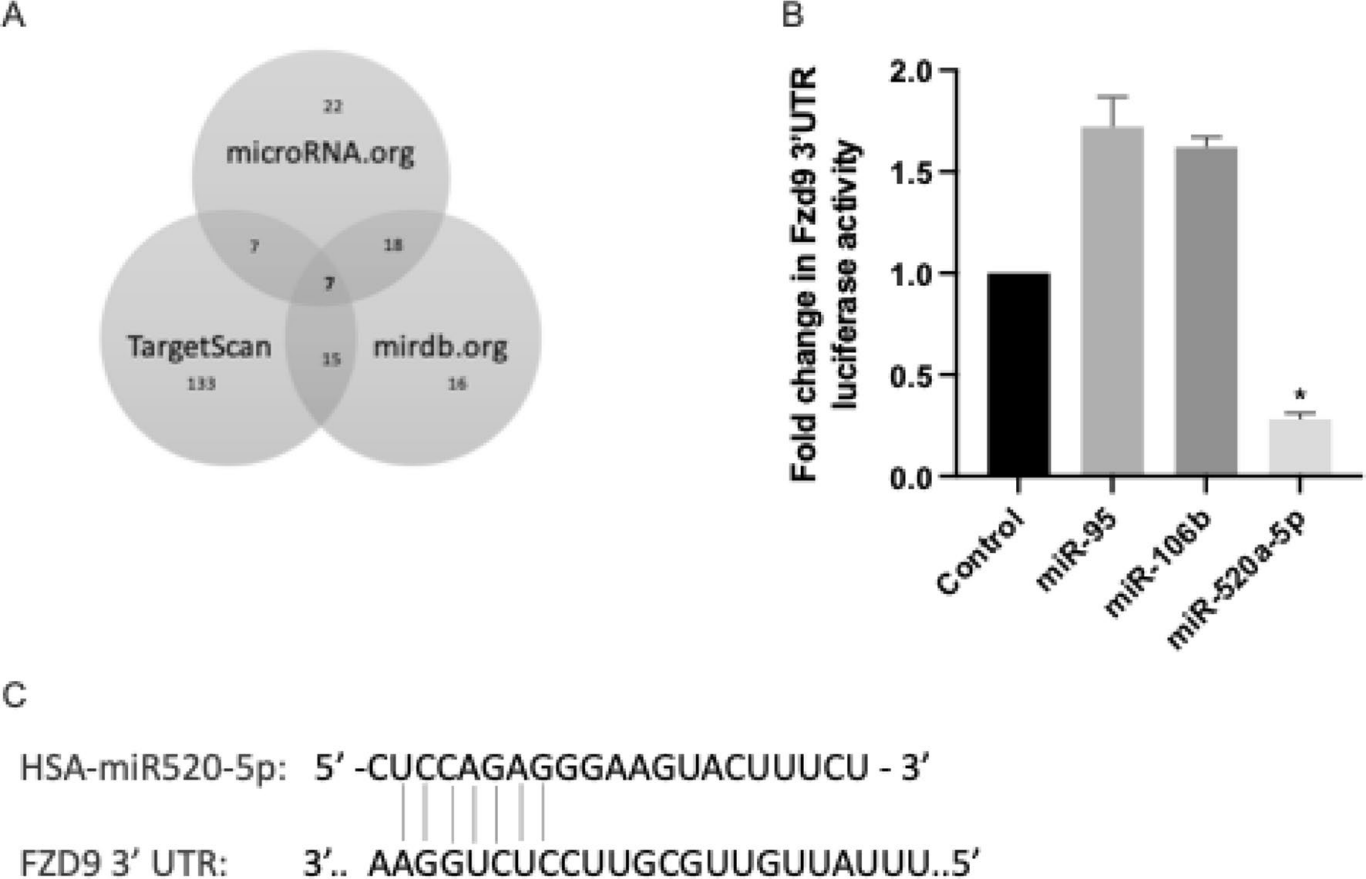
miRNA regulation of FZD9. (**A**) Results from database searches for miRNA predicted to target the FZD9 3’UTR. (**B**) miRNA mimics were transfected in triplicate into the A549 cell line with a FZD9 3’UTR luciferase. Luciferase activity was measured in duplicate and shown relative to control. *p < 0.05. (**C**) Alignment of miR520a-5p with the FZD9 3’UTR sequence.

**Figure 2:**
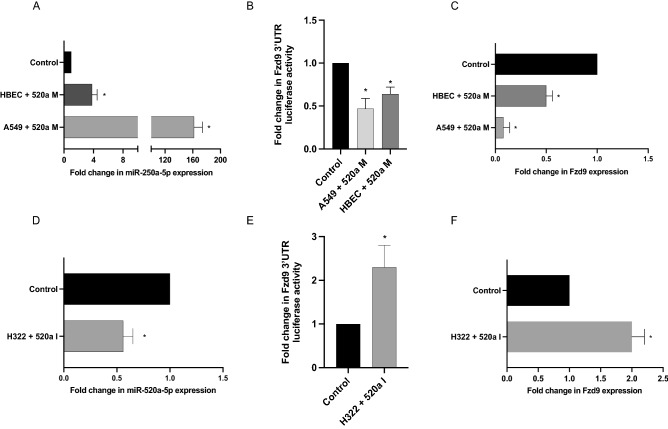
miR-520a-5p inhibits activity of the FZD9 3’UTR. miR-520a-5p expression (**A**), FZD9 3’UTR luciferase activity (**B**), and FZD9 expression (**C**) in FZD9 positive HBEC and A549 cell lines after transfection of a miR-520a-5p mimic and control. miR-520a-5p expression (**D**), FZD9 3’UTR luciferase activity (**E**), and FZD9 (**F**) in FZD9 negative H322 cell line after transfection of a miR-520a-5p inhibitor and control. Luciferase activity was measured in duplicate and shown relative to a control for each cell line. miRNA and mRNA expression were measured in triplicate by qPCR, normalized to RNU6 or GAPDH, and shown relative to control for each cell line. 520a M, miR-520a-5p mimic; 520a I, miR-520a-5p inhibitor. *p < 0.05.

We interrogated online miRNA databases to identify miRNA commonly predicted to bind to the FZD9 3’UTR and found seven miRNA that overlapped between the three databases (Fig. 1A). Based on confidence scores across the databases, we selected three miRNAs to initially test and found that the miR520a-5p mimic had the strongest effect on activity of a FZD9 3’UTR luciferase transfected into A549 cells (Fig. 1B). Figure 1C shows the alignment of miR520a-5p with the FZD9 3’UTR sequence. We elected to continue investigations on the role of miR-520a-5p in regulating FZD9 expression, with the expectation that other miRNA may also contribute to FZD9 regulation. We used A549 NSCLC cells because they have high levels of FZD9, are easily transfected, and allow investigation of FZD9 regulation in a tumor context^4^. The HBEC cell line is an immortalized, non-transformed cell line with moderate FZD9 expression, which we selected so we could test FZD9 regulation in a cell line more similar to lung cells that might be exposed to cigarette smoke in humans. When the miR-520a-5p mimic was transiently transfected into FZD9 positive A549 and HBEC cell lines, expression of miR-520a-5p increased (Fig. 2A). When co-transfected with the FZD9 3’UTR luciferase plasmid, luciferase activity decreased in both cell lines compared to the negative mimic control (Fig. 2B). FZD9 mRNA expression also decreased with transfection of the miR-520a-5p mimic in both A549 and HBEC (Fig. 2C). We selected H322 as an additional NSCLC line for FZD9 regulation tests based on its low expression of FZD9 and higher expression of miR-520a. In H322 cells, transient transfection with an inhibitor of miR-520a-5p decreased expression of miR-520a-5p (Fig. 2D) and increased activity of the FZD9 3’UTR luciferase (Fig. 2E). FZD9 expression in H322 increased with transfection of the miR-520a-5p inhibitor (Fig. 2F). In both lung epithelial and tumor cell lines, miR-520a-5p represses activity of the FZD9 3’UTR. In cells with FZD9 expression, the miR-520a-5p mimic also reduced FZD9 mRNA expression, while the miR-520a-5p inhibitor increased FZD9 mRNA expression in a FZD9 negative cell line.

### miR-520a-5p expression inhibits downstream function of FZD9

**Figure 3:**
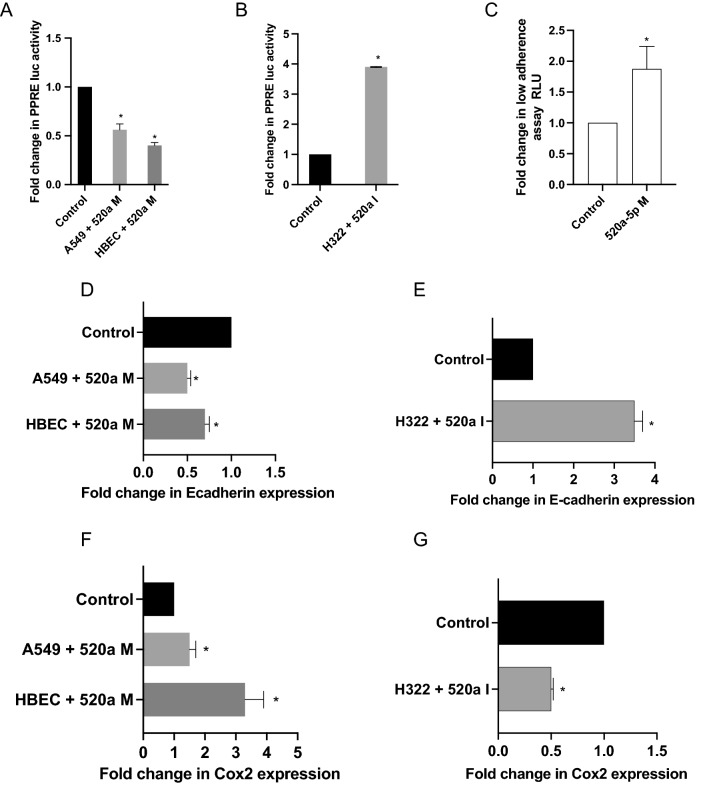
miR-520a-5p expression inhibits downstream function of FZD9. In A549 and HBEC (**A**) and H322 (**B**) cell lines, PPRE luciferase activity was measured after transfection of miR-520a-5p mimic or inhibitor and control. (**C**) A549 cells were transfected with miR-520a-5p mimic or control, cultured on a low-adherence plate for 48 h, and growth measured by fluorescent assay. After miR520a-5p mimic or inhibitor and control transfection, E-cadherin expression was measured in A549 and HBEC (**D**) and H322 (**E**) cell lines and Cox2 expression was measured in A549 and HBEC (**F**) and H322 (**G**) cell lines. Transfections were conducted in triplicate. Luciferase activity was measured in duplicate and shown relative to control for each cell line. mRNA expression was measured in triplicate by qPCR, normalized to GAPDH, and shown relative to control for each cell line. 520a M, miR-520a-5p mimic; 520a I, miR-520a-5p inhibitor. *p < 0.05.

When bound by its endogenous ligand Wnt7a or stimulated by iloprost, FZD9 activates PPARγ signaling^2^. To test the impact of miR-520a-5p on downstream targets of FZD9, we transiently transfected cells with a PPARG Response Element luciferase (PPRE). In FZD9 positive A549 and HBEC cells, the miR-520a-5p mimic decreased activity of PPRE (Fig. 3A). Transient transfection of a miR-520a-5p inhibitor into FZD9 negative H322 cells increased PPRE activity (Fig. 3B). To test the effect of miR520a-5p on transformed growth, we transfected A549 cells with the miR520a-5p mimic and measured growth in a low-adherence plate compared to control. Overexpression of miR-520a-5p led to a significant increase in A549 cell transformed growth in a low adherence assay (Fig. 3C). FZD9 alters expression of downstream targets of PPARG, such as E-cadherin and Cox2^4,10^. Transfection of a miR-520a-5p mimic led to inhibition of E-cadherin mRNA expression in A549 and HBEC cells and transfection of a miR-520a-5p inhibitor increased expression of E-cadherin in H322 cells (Fig. 3D,E). The miR-520a mimic increased Cox2, while the miR-520a-5p inhibitor decreased Cox2 (Fig. 3F,G). Targets of FZD9 have altered expression and function with manipulation of miR-520a-5p expression, indicating that binding of miR-520a-5p to the FZD9 3’UTR reduces the levels of FZD9 and inhibits downstream activity.

### CSC and iloprost alter miR520a-5p expression and FZD9 3’UTR activity

**Figure 4:**
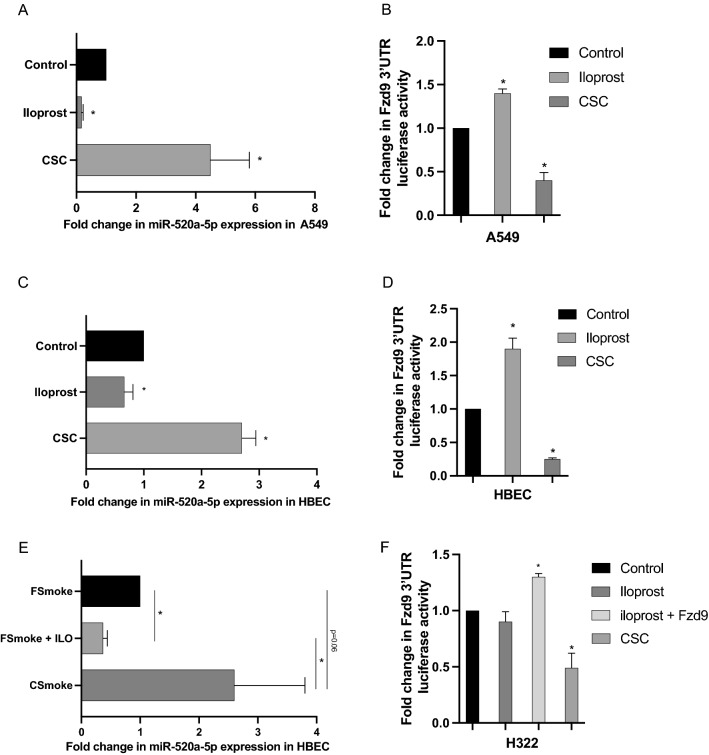
CSC and iloprost alter miR-520a-5p expression and FZD9 3’UTR activity. miR-520a-5p expression (**A**, **C**) and transfected FZD9 3’UTR luciferase activity (**B**, **D**) in A549 and HBEC cell lines after 48 h treatment with 5 µg/ml (HBEC) or 20 µg/ml (A549) CSC or 10 µM iloprost. (**E**) miR-520a-5p expression in the HBEC cell line treated with four weeks of 5 µg/ml CSC, followed by four weeks of removal of CSC (FSmoke), removal of CSC and addition of 10 μM iloprost (FSmoke + ILO), or continued CSC (CSmoke). (F) FZD9 3’UTR luciferase activity in the H322 cell line after transfection with FZD9 plasmid and 48 h treatment with 20 µg/ml CSC or 10 µM iloprost. Luciferase activity was measured in duplicate and show relative to control. miRNA expression was measured in triplicate by qPCR, normalized to RNU6, and shown relative to control. *p < 0.05 versus control.

FZD9 expression is decreased by CSC and increased by iloprost in lung epithelial cells in vitro and by cigarette smoke carcinogens and prostacyclin *in vivo*^10^. We investigated the effects of CSC and iloprost on miR-520a-5p and activity of the FZD9 3’UTR in lung epithelial cells and NSCLC cells. In FZD9 positive cells, 48 h of 20ug/ml (A549) or 5ug/ml (HBEC) CSC exposure increased miR-520a-5p expression (Fig. 4A,C) and decreased transfected FZD9 3’UTR activity (Fig. 4B,D). In HBEC, 48 h treatment with 10 µM iloprost decreased miR-520a-5p expression and increased activity of the FZD9 3’UTR (Fig. 4C,D). A549 cells also had a decrease in miR-520a-5p expression and increase in FZD9 3’UTR activity with 48 h of 10 µM iloprost treatment (Fig. 4A,B). In the oral iloprost chemoprevention clinical trial, former smokers benefitted from 6 months of iloprost treatment, but current smokers did not^11^. To mimic the trial in vitro, we exposed HBEC cells to CSC or vehicle for four weeks, then split the cultures into four weeks of continued CSC exposure (CSmoke), suspended CSC exposure (FSmoke), and suspended CSC exposure with iloprost treatment (FSmoke + ILO). miR-520a-5p expression in this experiment was higher in CSmoke cells compared to FSmoke cells and adding iloprost to FSmoke cells reduced expression of miR-520a-5p (Fig. 4E). In FZD9 negative H322 cells, 48 h of 20 µg/ml CSC reduced transiently transfected FZD9 3’UTR activity (Fig. 4F). As expected in a FZD9 negative cell line, treatment of H322 cells with 48 h of 10 µM iloprost alone did not alter FZD9 3’UTR activity because FZD9 is required for effects of iloprost in lung epithelial cells. However, when iloprost was combined with transfection of a FZD9 expression plasmid, the pathway was reestablished and iloprost increased FZD9 3’UTR activity (Fig. 4F). CSC and iloprost alter FZD9 mRNA levels and this data suggests they also modulate miR-520a-5p expression and subsequent activity of the FZD9 3’UTR.

### Iloprost and miRNA inhibition rescue effects of miR520a and CSC

**Figure 5:**
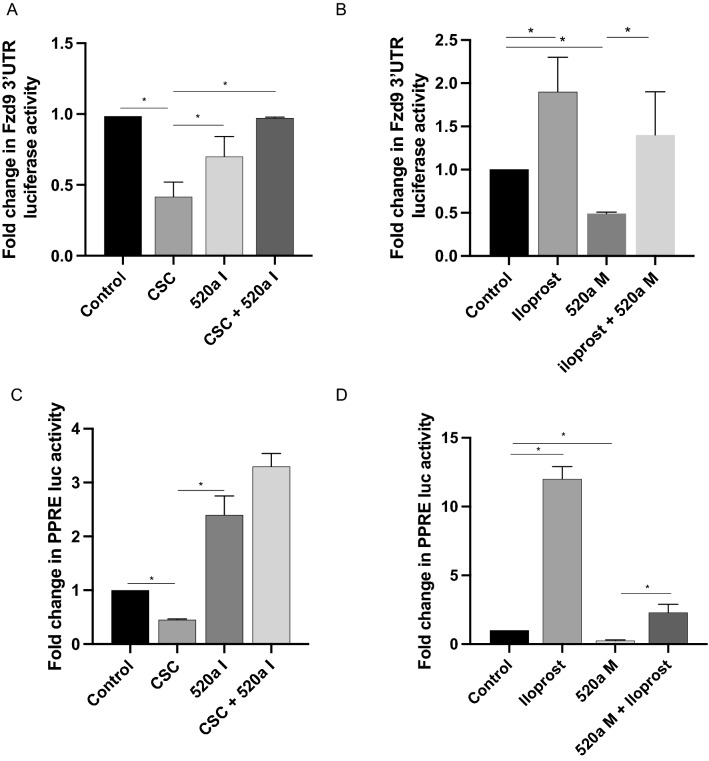
Iloprost and miR-520a-5p inhibition rescue the effects of miR-520a expression and CSC. Transfected FZD9 3’UTR activity (**A**) and PPRE activity (**C**) in the A549 cell line with 48 h of 20 µg/ml CSC exposure and/or a miR-520a-5p inhibitor. Transfected FZD9 3’UTR luciferase activity (**B**) and PPRE luciferase activity (**D**) with 48 h of 10 μM iloprost treatment and/or a miR-520a mimic. Luciferase activity was measured in duplicate and shown relative to control. 520a I, miR-520a-5p inhibitor; 520a M, miR-520a-5p mimic. *p < 0.05.

We used rescue experiments in A549 cells as proof of concept for the relationship between CSC and iloprost exposure, miR-520a-5p, and FZD9. Inhibiting miR-520a-5p during CSC exposure reversed the effects of CSC on the FZD9 3’UTR luciferase, returning activity to control level (Fig. 5A). Treating with iloprost partially reversed the effects of the miR-520a-5p mimic on FZD9 3’UTR activity (Fig. 5B). Downstream signaling could also be rescued, with inhibition of miR-520a-5p reversing the effects of CSC on PPRE (Fig. 5C) and iloprost treatment reversing the effects of the miR520a-5p mimic on PPRE, although the miR-520a-5p mimic appeared to limit the magnitude of iloprost’s stimulation of PPRE (Fig. 5D). Both the miR-520a-5p inhibitor and mimic appear to have a stronger effect on PPRE luciferase activity compared to FZD9 3’UTR luciferase activity, suggesting that there may be additional pathways inhibited by miR-520a-5p that activate PPARG signaling.

## Discussion

With this study, we discovered the first miRNA to regulate the transmembrane receptor FZD9 in lung epithelial cells and demonstrated its role in the effects of CSC and iloprost on FZD9. We found that miR-520a-5p inhibits activity of the FZD9 3’UTR and also leads to decreased FZD9 mRNA expression. Function of FZD9, as measured by low adherence assay and PPRE activity, was decreased by expression of miR-520a-5p, as was expression of established FZD9 target genes in the lung. In a FZD9 negative NSCLC cell line, inhibition of miR-520a-5p increased activity of the FZD9 3’UTR, increased FZD9 function and downstream target expression, and increased FZD9 mRNA expression. FZD9 expression is required for iloprost activity and is decreased with CSC exposure in vitro and in vivo, so we tested whether miR-520a-5p played a role in these effects^4,10^. We found that CSC increased miR-520a-5p expression in both a FZD9 positive NSCLC cell line and a normal bronchial epithelial cell line. CSC also decreased activity of the FZD9 3’UTR, demonstrating that induction of miR-520a-5p is a direct mechanism of cigarette smoke induced FZD9 repression. Various carcinogens, including cigarette smoke, have been shown to alter miRNA expression levels in cancer^12^. The most described mechanism by which carcinogens contribute to differential miRNA expression is through epigenetic alterations^13,14^. Other observed mechanisms include adduct changes within the nucleotides of both miRNA and DNA and alternative expression or posttranslational modification of the dicer miRNA processing protein^15–17^. Further studies of miR-520a-5p in carcinogenesis and premalignant lung lesions could clarify the specific mechanism that leads to altered expression.

**Figure 6:**
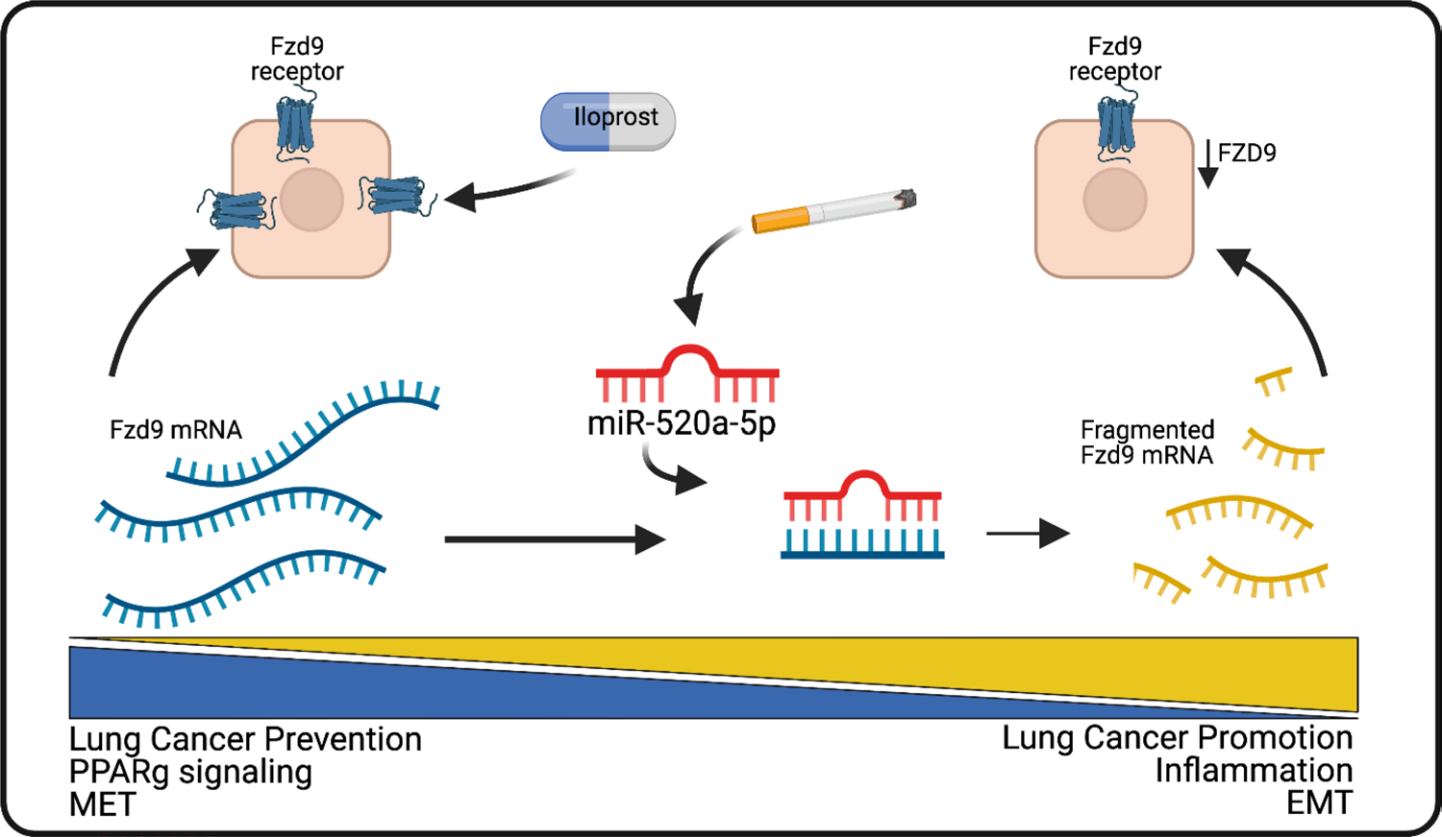
miR-520a-5p regulates FZD9. miR-520a-5p binding to the FZD9 3’UTR region reduces translation of the receptor, increases cancer promoting signaling, and interferes with response to iloprost. With at least some presence of the FZD9 receptor, iloprost can overcome increased miR-520a-5p expression to intercept progression of premalignant lesions in the lung. Figure designed with BioRender.

Iloprost decreased expression of miR-520a-5p and increased FZD9 3’UTR activity, supporting inhibition of miR-520a-5p as a mechanism for iloprost in lung epithelial cells and suggesting a feedback loop where iloprost amplifies the expression and activity of the receptor required for its chemopreventive effect. Inhibition of miR-520-5p reversed effects of CSC on FZD9 3’UTR and downstream PPRE activity, while expression of miR-520a-5p inhibited the effects of iloprost. The strong effect of the miR-520a-5p mimic and inhibitor on PPRE luciferase activity suggests more work is needed to determine additional PPARG targeting pathways that are inhibited by this miRNA. miR-520a-5p is not conserved, so we could not conduct studies in mouse tissue and will need to explore expression further in human tissues. This study was also limited by poorly performing existing FZD9 antibodies that do not reliably detect FZD9-specific expression in lung cells, so transformed growth and downstream activity of PPRE were included as measures of FZD9 protein function. In lung epithelial cells, we propose a system where miR-520a-5p contributes to regulation of FZD9 expression (Fig. 6). The cancer promoting effects of FZD9 loss after cigarette carcinogen exposure are mediated by miR-520a-5p and the lung cancer chemoprevention agent iloprost acts in part by reversing the effects of miR520a-5p.

In lung cancer cells, Frizzled 1 can be inhibited by miR-135b, Frizzled 2 by miR-203, Frizzled 4 by miR-3127-5p and miR-204, Frizzled 5 by miR-29a, and Frizzled 7 by miR-27-3, generally leading to inhibition of oncogenic Wnt/β-catenin signaling^18–23^. miRNA have also been identified that inhibit other parts of the Wnt/β-catenin signaling pathway in lung cancer, such as SFRPs, but these studies predominantly demonstrate indirect regulation of Wnt/β-catenin signaling^24,25^. FZD9 is unique among Frizzled receptors in that it does not activate oncogenic signaling pathways in lung cancer but contributes to maintenance of a normal lung epithelium and exerts tumor suppressive effects when partnered with Wnt7a or iloprost^1,2,4^. miR-31 indirectly affects FZD9 expression to mediate the effects of prostacyclin and CSC exposure in human bronchial epithelial cells (HBEC), non-small cell lung cancer (NSCLC) cell lines, and mice^10^. However, miRNA that directly target FZD9 have not been described in any context prior to our current study, so this work represents an important step toward understanding regulation of FZD9 in lung cancer.

There are limited published studies on miR-520a and most investigate activity of miR-520a-3p. miR-520a expression has been associated with deleterious states, such as obesity, esophageal carcinoma, preterm birth, preeclampsia, and RSV infection^26–30^. In cancer studies, miR-520a has been associated with suppression of oncogenic signaling pathways including CDK4, SUV39H1, LIMK1, GOT-2, AKT1/mTOR, and PI3K/AKT^31–37^. miR520-3p expression inhibits proliferation in vitro and inhibits tumor growth in xenograft experiments for several cancer types^32,38–43^. Two studies have also shown that miR-520a-5p inhibits lung tumor cell proliferation^31,34^. While previous manipulations of miR-520a in vitro and in vivo portray an oncogenic miRNA, in the OncoMir Cancer Database (https://www.oncomir.umn.edu/omcd/), miR-520a-3p/5p expression in normal human tissue compared to either lung adenocarcinoma or lung squamous carcinoma is not significantly different. In the dbDEMC cancer miRNA database (https://www.picb.ac.cn/dbDEMC/), miR-520a-3p expression trends differently in lung depending on the study. We focused on the A549 NSCLC cell line in rescue experiments because it is a dependably transfectable cell line with high FZD9 expression that was amenable to use of a miR-520a mimic and inhibitor. The A549 experiments supported our primary hypothesis, as did data from the HBEC cell line, which represents a context in which miR-520a has not been previously investigated. While previous studies may contrast with our data by suggesting that miR520a-5p would act like an tumor suppressor in the context of lung tumors, there may be different roles for miR-520a depending on the stage of lung lesion or degree of aberrant signaling. As demonstrated in a study of multiple miRNAs over the course of bronchial lesion development, miRNA expression can increase and decrease, suggesting that miR-520a-5p expression and function could fluctuate from CSC exposure to advanced tumor development^44^. In lung cells, miR-520a-3p appears to be sponged by lncRNA and circ-RNA, suggesting additional layers of potential regulation in the FZD9, iloprost, and cigarette smoke pathway^31,39,40,43^. Future studies could address this complexity and confirm the FZD9-miR520a connection by profiling expression across a spectrum of premalignant lung lesions and lung tumors.

We previously showed that iloprost increases FZD9 mRNA expression and now show that the decrease of a post-transcriptional repressor of FZD9 also leads to increased FZD9 mRNA expression^10^. Potential feedback loops for FZD9 have not yet been explored, but the FZD9 promoter region has a potential binding sequence for PPARG, suggesting that induction of FZD9 expression by iloprost may occur through the transcription factor function of PPARG and its additional effects on miR-520a. Pre-or post-transcriptional regulators of FZD9 in the adult lung are unknown, so this data provides potential new research directions. Future studies will clarify the activity of miR-520a in multiple cell and malignancy contexts to determine how miR-520a plays a pro- or anti-tumor role in the lung. There are likely multiple miRNA regulators of FZD9 and with this proof of concept, studies of additional miRNA important for FZD9 activity will be conducted. Additional emerging data on Fzd receptors suggest they are better targets than previously thought for small molecule agents, further supporting future studies of iloprost and FZD9 interaction^45,46^. Our results show that miR-520a-5p targets FZD9, identifying a new method of FZD9 regulation induced by cigarette smoke that could impact iloprost’s activation of anti-cancer signaling pathways. This work improves our understanding of FZD9 regulation in the lung and provides valuable insight for future studies of the mechanisms of iloprost lung cancer chemoprevention.

## Methods

*Predicted miRNA screen*: We interrogated three online databases (Targetscan, microRNA.org, and mirdb.org) to find miRNA commonly predicted to bind to the FZD9 3’UTR. FZD9 was input as the human gene symbol and lists of miRNAs predicted to target FZD9 were compared between databases.

*Cell Culture:* Non-transformed human bronchial epithelial cells (HBEC3KT) (a gift from the lab of Dr. John Minna, UT Southwestern) were cultured in Keratinocyte Serum Free Medium (GIBCO) at 37 °C in a humidified 5% CO_2_ incubator and passaged twice per week. All HBEC cell cultures were grown and handled in a dedicated incubator. A549 and H322, human lung cancer cell lines (purchased from the Tissue Culture Core Facility at the University of Colorado Cancer Center), were cultured in RPMI (GIBCO) with 10% Fetal Bovine Serum at 37 °C in a humidified 5% CO_2_ incubator and passaged twice per week. To generate cigarette smoke condensate (CSC), filters from a TE-10 smoking machine (Teague Enterprises) were weighed before and after smoking ten cigarettes and then soaked in DMSO to recover cigarette smoke particulate. The resulting condensate was used in cell growth media at 5 or 20 ug/mL with DMSO as a vehicle control. Iloprost (Cayman Chemicals) was used at 10 µM in cell growth media with methyl acetate as a vehicle control. Cells with CSC and iloprost exposure were carried in triplicate and treated every 24 h, with 24 h to recover from passaging. For the former smoke HBEC experiment, HBEC were continuously cultured in triplicate for four weeks with or without CSC. Cultures were then split into triplicate plates with continued CSC exposure, no CSC exposure, or no CSC with 10 µM CSC. Cells were cultured with these conditions for another four weeks and then RNA was harvested^10^.

*Transfections:* Cells were transfected in triplicate with mimics for hsa-miR-95-5p, hsa-miR-106b, or hsa-miR-520a-5p mimic (2.5 nM)(Qiagen), negative mimic control (2.5 nM)(Qiagen), hsa-miR-520a-5p inhibitor (25 nM) (Qiagen), negative inhibitor (25 nM)(Qiagen), miRNA FZD9 3’ UTR Target Clone (50 ng) (GeneCopoeia), PPARγ response element luciferase (a gift from Bruce Spiegelman; Addgene plasmid #1015) (50 ng), renilla control reporter vector (Promega) (25 ng), and/or FZD9 expression plasmid (GeneCopoeia) (25 ng) using 0.25 ul TransIT-X2 transfection reagent (Mirus Bio) per the manufacturer’s protocol. Transfections were analyzed after 48 h. Transfection data is representative of triplicate experiments and included empty and mock transfection controls. Significance was assessed by T-test or ANOVA in GraphPad Prism. For the transformed growth assay, A549 cells were transfected with the miR520a-5p mimic or negative mimic control as described above. At 24 h, cells were moved to a low-attachment plate (S-BIO) at a concentration of 1000 cells/well. At 72 h, cell growth was analyzed using the CellTiter Glo Assay (Promega)^47^.

*Luciferase assays:* FZD9 3’UTR luciferase activity was measured using the Secret-Pair Dual Luminescence Assay kit (GeneCopoeia) for parallel bioluminescence assays of secreted gaussia luciferase and alkaline phosphatase on a Glomax instrument (Promega). 3’ UTR luciferase assays were measured in duplicate, and data is representative of triplicate experiments. Activity from PPRE and renilla control luciferase plasmids was measured using the Dual-Luciferase Reporter Assay Kit (Promega) on a Glomax instrument. PPRE assays were measured for triplicate experiments. Significance was assessed by T-test or ANOVA in GraphPad Prism.

*Quantitative PCR:* RNA was extracted from cell lines using the RNeasy Plus kit (Qiagen). Total RNA was reverse transcribed using the miScript II RT kit (Qiagen). miScript primer assays (Qiagen) were used for hsa-miR-520a-5p and RNU6 miRNA and Prime PCR Assays (Bio-Rad) for FZD9 and GAPDH. qPCR was conducted with the miScript qPCR kit (Qiagen) or Sso Advanced SYBR Green Master Mix (Bio-Rad) on a CFX 96 Touch (Bio-Rad). All PCR reactions were conducted in triplicate and data presented is representative of experiments conducted in triplicate*.* All gene expression data was normalized to GAPDH or RNU6 and fold changes were calculated using the 2^−ΔΔCt^ method. Significance was assessed by T-test or ANOVA in GraphPad Prism.

## Data Availability

No datasets were generated or analyzed during the current study. Any requests for information about access to methods, materials, or data can be addressed to the corresponding author.
